# Massive hemoperitoneum from hemorrhagic corpus luteum in a patient with acquired amegakaryocytic thrombocytopenic purpura

**DOI:** 10.1002/ccr3.2767

**Published:** 2020-03-07

**Authors:** Esther Ifeoma Obi, Nkencho Osegi, Theodora Phiemueki Etu‐Efeotor, Ogho Crosdale Pughikumo, Helen Toluhi, Olakunle Makinde

**Affiliations:** ^1^ Department of Haematology Federal Medical Centre Yenagoa Nigeria; ^2^ Department of Obstetrics and Gynaecology Federal Medical Centre Yenagoa Nigeria; ^3^ Department of Haematology and Immunology College of Health Sciences Niger Delta University Wilberforce Island Nigeria

**Keywords:** amegakaryocytic thrombocytopenic purpura, corpus luteum, corpus luteum hemorrhagium, hemoperitoneum, immune thrombocytopenic purpura, thrombocytopenia

## Abstract

Doctors should think of a spectrum of differential diagnoses in patients presenting with acute abdominal pain ranging from medical, surgical, and gynecological conditions. Proper laboratory and radiological tests including bone marrow study are advised.

## Graphical abstract

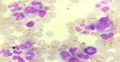

## 1 INTRODUCTION

Amegakaryocytic thrombocytopenic purpura (AATP) is an uncommon hematological disorder characterized by thrombocytopenia resulting from an unexplained reduction in the number of bone marrow megakaryocytes in the presence of otherwise normal hematopoiesis.^1^, ^2^ This is in contrast to immune thrombocytopenic purpura (ITP) typified by hyperplasia of megakaryocytes in the bone marrow.^1^

Corpus luteum is a temporary endocrine structure formed during luteinization of the follicle after ovulation. Corpus luteum hemorrhage may be caused spontaneously or triggered by coitus, trauma, exercise, or vaginal examination. Thus, the risk of hemorrhagic ruptured corpus luteum may be increased in other hemostatic disorders such as ITP, hemophilia, and AATP.^3^

We describe therein a case of AATP who had spontaneous massive hemoperitoneum from corpus luteum hemorrhage as part of her presentation.

## 2 CASE REPORT

**Figure 1:**
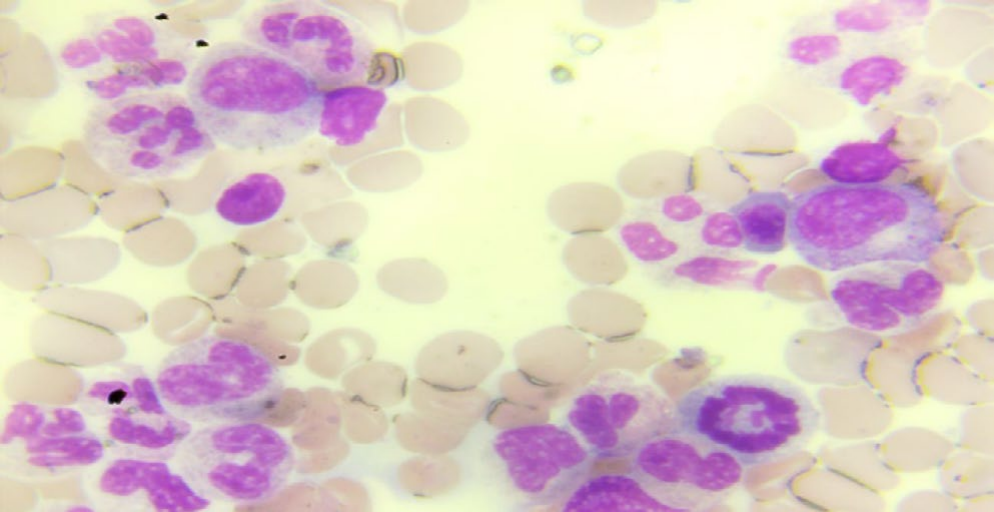
Normal erythropoiesis and myelopoiesis with virtually no megakayocyte seen

**Figure 2:**
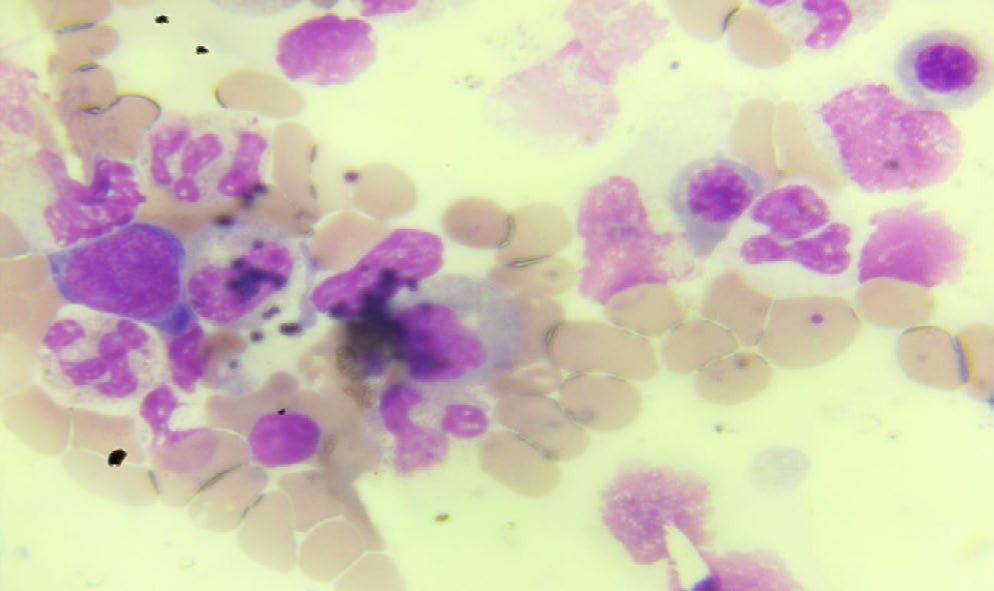
Bone marrow film showing absent megakaryocytes with normal haematopoiesis of other cell lines

A 20‐year‐old female presented to the emergency department with one‐week history of recurrent nose and gum bleeds, passage of bloody urine, and generalized abdominal pain of three days’ duration. Abdominal pain started in the right lower quadrant, the pain gradually became generalized and increased in severity. Past medical history did not reveal any bleeding disorder or use of anticoagulant therapy. The patient had stable vital signs on physical examination with a pulse rate of 108 beats per minute, a blood pressure of 110/50 mm Hg, and a temperature of 36.8°C. Positive findings on physical examination were severely pale conjunctiva, generalized petechial hemorrhage, purpural hemorrhage on the medial aspect of the left thigh, and generalized abdominal tenderness. She had no abdominal guarding or rebound tenderness. Important paraclinic results were hemoglobin 5.8 g/dL, platelets 3.9 × 10^9^/L, international normalized ratio 1.4, partial thromboplastin time 52 seconds, fibrinogen 158.4 mg/dL, and D‐dimer 2360.5 ng/mL. Peripheral blood smear showed reduced platelets. Pregnancy test was negative. Free intraperitoneal fluid collection was reported on abdominopelvic ultrasound scan. Emergency exploratory laparotomy and right salpingo‐ovariectomy done revealed massive hemoperitoneum 3.5 L. Patient was managed pre‐, intra‐, and postoperatively with fresh whole blood transfusions and platelet concentrates. She received a total of 16 units of fresh whole blood transfusions. There was minimal rise in the platelet count despite these transfusions, not greater than 5.9 × 10^9^/L. A working diagnosis of immune thrombocytopenia was made, and the patient was managed with steroids: high dose of methylprednisolone at 2 mg/kg for 3 days continued with oral prednisolone 1 mg/kg. Bone marrow aspiration done showed virtually no megakaryocytes with other cell lines appearing normal as shown in Figures 1 and 2. Histology report of the surgical specimen revealed corpus luteum hemorrhagium.

A definitive diagnosis of corpus luteum hemorrhagium from acquired amegakaryocytic thrombocytopenic purpura was made.

Patient did not respond to prednisolone after a month therapy and bleeding symptoms unabated. A second‐line drug, oral mycophenolate mofetil (MMF), an immunosuppressant was included. She received a partial response with a rise in the platelet count to 41 × 10^9^/L and bleeding symptoms like the persistent hematuria and ecchymoses resolved. Hemoglobin concentration rose to 9.5 g/dL.

She is presently on oral MMF 1 g, and platelet count has remained above 50 × 10^9^/L after three months.

## 3 DISCUSSION

Amegakaryocytic thrombocytopenic purpura, though very rare, should be considered in any case of thrombocytopenia that does not respond to the usual line of treatment, as in our case. ITP promptly responds to steroids, which is not the case in AATP as seen in the index case.^2^, ^4^

The prevalence of AATP is unknown as most cases are not diagnosed or misdiagnosed as ITP. Both cell‐mediated immunity and humoral immunity play a role in causation of this condition, some authors view that AATP may be the result of ineffective thrombopoiesis caused by the auto antibodies leading to suppressed megakaryocyte production.^4^, ^5^

In the assessment of abdominal pain in women, it is of great importance that physicians consider special differential diagnoses related to gynecological disorders such as corpus luteum rupture.^3^

The female patient in reproductive age, with inherited bleeding disorder have twice higher incidence of hemorrhagic ovarian cysts in comparison to the general population. These cysts are due to excessive bleeding into the corpus luteum at the time of ovulation, and rupture of these cysts may result in hemoperitoneum. The prevalence of spontaneous massive hemoperitoneum due to hemorrhagic corpus luteum cyst is extremely rare, but it can be a potentially life‐threatening presentation. Corpus luteal bleeding is described more from the right ovary as it is believed that the recto‐sigmoid colon helps protect the left ovary from trauma, and there is higher intraluminal pressure on the right side because of the difference in ovarian vein architecture.^3^, ^6^, ^7^

Corpus luteum hemorrhage can be a diagnostic dilemma and diagnosis is seldom made preoperatively. In milder cases, the patients may be subjected to surgery on suspicion of acute appendicitis or in severe cases ectopic pregnancy as seen in the index case.

Treatment of AATP is not clearly defined. It usually depends on the availability, cost factors, and side effects of drugs. It is advisable to start with the least toxic and cost‐effective therapy with periodic monitoring of treatment responses. A change in therapy will be required in treatment failure.

Immunosuppressive agents have a role in treating this condition. There are reports of AATP being treated as monotherapy or in combination with antithymocyte globulin (ATG), azathioprine, danazol, mycophenolate mofetil (MMF), and rituximab with varying degrees of success. More aggressive approach with myeloablative chemotherapy followed by allogenic bone marrow transplantation has also been reported to be successful.^8^, ^9^

## CONFLICT OF INTEREST

None declared.

## AUTHOR CONTRIBUTION

EIO: analyzed and interpreted the patients’ data regarding the hematological disease and was a major contributor in writing the manuscript. NO: analyzed and interpreted the patients’ data regarding the gynecological disease and review of the manuscript. TE and CP: performed the morphological examination of the peripheral blood film/ marrow aspirate and producing the images used in the manuscript. HT and OM: contributed in literature review and writing the discussion. All authors read and approved the final manuscript.

## References

[1] Pakra MA , Jabri AA , Hanafy E . Myelodysplastic syndrome presenting as amegakaryocytic thrombocytopenia in a collodion baby. J Invest Med High Impact Case Rep. 2015;3(3):1‐3. 10.1177/2324709615605637 PMC4748500 26904703

[2] Abdulsalam MS , Vijayanarayanan A , Pandurangan P , Soni M , Katchabeswaran R . Acquired amegakaryocytic thrombocytopenic purpura with literature review. J Appl Haematol. 2017;8:116‐118.

[3] Sayyed MS , Azra I , Morteza TD , Azadeh MG . Hemorrhagic corpus luteum with generalized abdominal pain in patients with idiopathic thrombocytopenic purpura. Razavi Int J Med. 2016;4(1):e34533.

[4] Betdur AL , Bhagra A , Tej K , Ponnambalam R , Somasekar DS An unusual case of amegakaryocytic thrombocytopenia. J Evid Based Med Healthcare. 2014;1(17):2222‐2226.

[5] Hoffman R , Bruno E , Elwell J , et al. Acquired amegakaryocytic thrombocytopenic purpura: a syndrome of diverse etiologies. Blood. 1982;60:1173‐1178. 6982086

[6] Ikram N , Zafar T , Case AS . Series Haemorrhagic ovarian cysts in females with inherited bleeding disorders. JRMC. 2009;13(1):48‐50.

[7] Gandhi R , Bahri N , Parekh H , Chuasama S , Doshi N , Muniya C . Ovarian torsion with ruptured ovarian haemorrhage with massive hemoperitoneum in a case of ITP. Int J Radiol. 2009;11(2):6.

[8] Zhang WG , Ji L , Cao XM , et al. Mycophenolate mofetil as a treatment for refractory idiopathic thrombocytopenic purpura. Acta Pharmacol Sin. 2005;26(5):598‐602. 15842780 10.1111/j.1745-7254.2005.00088.x

[9] Bulchandani D , Nachnani J , Belt R , Hinton S . Acquired pure megakaryocytic aplasia: report of a single case treated with mycophenolate mofetil. Am J Haematol. 2007;7:605‐651. 10.1002/ajh.20895 17301968

